# Combined tDCS and Neuropsychological Treatment for Adult ADHD: A Single-Case Feasibility Study on Cognitive and Emotional Outcomes

**DOI:** 10.3390/brainsci16030339

**Published:** 2026-03-21

**Authors:** Pablo Rodríguez-Prieto, Julia Soler-Vázquez, Joaquín A. Ibáñez-Alfonso

**Affiliations:** 1 University Clinic of Psychology, Department of Psychology, Universidad Loyola Andalucia, 41704 Sevilla, Spain; prprieto@uloyola.es (P.R.-P.); juliasoler@ihppediatria.com (J.S.-V.); 2 Instituto Hispalense de Pediatría (IHP), 41014 Sevilla, Spain

**Keywords:** neuromodulation, tDCS, ADHD, anxiety, depression, emotional symptoms, executive functions, cognition, neurorehabilitation, psychotherapy

## Abstract

**Highlights:**

**What are the main findings?**

- The multimodal intervention combining anodal tDCS, digital neurorehabilitation, and psychotherapy showed preliminary clinical improvements in higher-order executive functions, including planning, cognitive flexibility, and visual working memory.
- Significant clinical reductions in depressive symptoms and measurable enhancements in physical and psychological quality of life domains were observed, suggesting the potential of the protocol for modulating emotional regulation in a clinical setting.

**What are the implications of the main findings?**

- Findings suggest a synergistic effect between prefrontal neuromodulation and behavioral interventions, providing a comprehensive framework to address the cognitive–emotional interplay in adult ADHD.
- The study establishes the safety and feasibility of integrated non-pharmacological protocols, offering a promising evidence-based alternative for adult patients who remain underrepresented in current treatment paradigms.

**Abstract:**

**Background/Objectives**: Attention Deficit Hyperactivity Disorder (ADHD) is one of the most common neurodevelopmental disorders in childhood and it tends to remain during adulthood. It not only affects cognitive abilities and behavior but also often presents emotional disturbances and alterations in the perceived quality of life. These symptoms are primarily related to dysfunctions in the ventromedial and dorsolateral prefrontal network. The main objective was to evaluate the feasibility and explore the initial outcomes of an integrated protocol combining neuropsychological treatment and transcranial direct current stimulation (tDCS). **Methods**: This study presents a single-case experimental A-B design of a 21-year-old woman, diagnosed with predominantly inattentive ADHD, treated at the University Psychology Clinic of Loyola Andalucía University. The treatment was carried out twice a week for 5 weeks (10 sessions in total), with 20 min of anodal tDCS at F3 and cathodal tDCS at F4 (2 mA), while digital neurorehabilitation exercises and psychotherapeutic support were provided. **Results**: An overall significant improvement was observed in cognitive functions (*p* = 0.008), with clinically significant gains in cognitive flexibility, visual working memory, and planning. Mixed results were found in inhibition, with improvement in interference control but no change in response inhibition. No significant changes were observed in sustained attention, auditory working memory, or processing speed. In terms of emotional state, an overall improvement was noted (*p* = 0.046), particularly in depression symptoms and perceived quality of life related to physical and psychological health. However, no significant changes were observed in anxiety symptoms or in areas related to the environment and social relationships. These findings reflect pilot-level evidence of clinical change within a feasibility framework. **Conclusions**: The combined treatment was found to be safe and feasible, showing promising preliminary improvements in cognitive and emotional domains. As a single-case study, these results serve as hypothesis-generating evidence for future controlled trials.

## 1. Introduction

Attention Deficit Hyperactivity Disorder (ADHD; code ICD-11: 6A05) originates in childhood and is characterized by a persistent lack of attention and/or hyperactivity–impulsivity, which causes impairment in different areas of daily life [1]. Although ADHD symptoms in adults are very similar to those in children, fundamental differences exist. For example, hyperactivity and impulsivity tend to decrease with age, unlike inattention [2]. Moreover, hyperactivity can manifest as internal tension or internal restlessness, leading to misdiagnoses such as anxiety [3]. ADHD is one of the most common brain development disorders in children, with an approximate global prevalence of 5% [4]. Longitudinal studies following children with ADHD over time show that between 30% and 60% experience persistent symptoms and/or functional difficulties in adulthood [5,6,7]. These findings are consistent with cross-sectional epidemiological studies indicating that the prevalence of ADHD in adults ranges between 2% and 4% [8,9]. Despite this high prevalence, there is a lack of diagnosis and adequate care for most adults with ADHD.

The cause of ADHD has been described as multifactorial and complex [10]. It is a condition that arises from the interaction between environmental and genetic factors [11]. In terms of genetic factors, ADHD has been shown to be a polygenic condition, implying the involvement of multiple genes and their corresponding variants such as the dopamine transporter gene (SLC6A3) and the dopamine receptor D4 (DRD4). While pediatric ADHD is strongly linked to these variants, adult ADHD polygenicity often involves broader neurodevelopmental and synaptic plasticity pathways [12,13]. Furthermore, adult ADHD is characterized by distinct neurophysiological disturbances. Electroencephalogram (EEG) studies frequently report an increased theta/beta ratio, particularly over frontal regions, which is often interpreted as a marker of cortical hypoarousal and impaired top-down inhibitory control, correlated with the executive deficits observed in the clinical population [14]. Regarding environmental factors, significant data has been obtained in recent years indicating that both prenatal and postnatal factors are associated with an increased risk of developing ADHD. During the prenatal period, factors such as maternal tobacco and alcohol use, low birth weight, premature birth, and exposure to environmental toxins can have an impact [15]. In the postnatal stage, a connection has been established between maternal postpartum depression and the presence of ADHD symptoms in their children [16]. Considering brain structure, anomalies such as a reduction in total brain size, especially gray matter, are observed [17,18,19]. A more pronounced decrease in volume is found in areas such as the prefrontal cortex, basal ganglia, and cerebellum, which is related to the severity of symptoms in individuals with ADHD [10,17,18], and these volume losses persist into adulthood [20]. Functional imaging studies reveal reduced activation patterns in areas such as the prefrontal cortex, striatal, and cerebellar regions [10,17,20,21]. From a neurochemical perspective, it is postulated that alterations in the dopaminergic and noradrenergic systems underlie ADHD symptoms, with a decrease in the availability of dopamine in the synapse and the importance of norepinephrine in the functioning of the frontal lobes involved in emotional and behavioral regulation [12,22,23].

Individuals with ADHD show deficits in executive functions (EFs). These functions are responsible for higher-order cognitive processes that allow for the proper regulation and adjustment of responses to achieve goals, especially in challenging or unfamiliar situations [24]. Working memory, processing speed, and inhibitory control are among the most affected [25,26,27]. The emotional profile in individuals with ADHD is also altered. Emotional regulation involves an interaction between the ventromedial and dorsolateral prefrontal cortex, which are two fundamental mechanisms linking emotion and cognition in humans [28,29]. This interaction is bidirectional: firstly, emotional stimuli influence EFs by assigning an emotional value to certain stimuli, which increases their likelihood of being selected for cognitive processing. Secondly, the existence of a regulatory function that controls the onset, duration, intensity, and content of the emotional response through executive functions is postulated. These brain areas establish a system for regulating activity in limbic structures such as the amygdala, hippocampus, and basal ganglia, thereby generating cortical regulation of emotional responses. Any deterioration in executive functions leads to deficits in emotional regulation, which manifests as a tendency to react intensely, also known as emotional lability, which is common in ADHD [30]. Furthermore, it is common for patients with this disorder to present comorbidity with depression and anxiety disorders. They are estimated to be three times more likely to develop anxiety disorders, while the risk of suffering from major depression or dysthymia is five times higher compared to individuals without ADHD [12].

The care approach for adult ADHD should be comprehensive, combining non-pharmacological interventions and, when necessary, pharmacological treatment [3]. Two primary goals exist: reducing functional impairment and improving symptoms. Psychoeducation, cognitive–behavioral therapy (CBT), and environmental adaptations aim to reduce impairment [3], while cognitive rehabilitation and medications address symptoms [31]. Stimulants enhance dopamine and norepinephrine levels, improving cognition and alleviating symptoms of inattention and hyperactivity–impulsivity [32]. They also improve negative emotional and behavioral symptoms in both children and adults [33]. Non-stimulants serve as alternatives for patients who do not respond to stimulants or experience adverse effects [12]. Medication initiation is considered when symptoms cause significant social and functional difficulties [34], but approximately 30% of patients show limited improvement with pharmacological treatment [12], highlighting the need for complementary therapies.

Non-pharmacological interventions include therapies based on cognitive–behavioral theory, such as traditional CBT, dialectical–behavioral therapy, and mindfulness-based therapies [35]. The treatment begins with psychoeducation, which involves providing information about the disorder and its impact on the patient’s life, benefiting both the patient and their family [36]. CBT, either individually or in groups, focuses on improving self-control, problem-solving, and social skills. Cognitive stimulation, another therapeutic strategy, involves exercises to enhance cognitive functions like attention, memory, and planning, using both traditional and technological tools [37]. Meta-analyses show moderate effectiveness in improving cognitive functions, with the recommendation to combine these interventions with psychoeducation, behavioral therapy, and pharmacological treatment as needed [38].

Another non-pharmacological treatment that is currently being proposed for ADHD is neuromodulation. It refers to therapeutic techniques that seek to modify the activity of brain structures through electrical, or magnetic stimuli, with the aim of treating various neuropsychiatric conditions [39,40]. These techniques are based on the idea that selective stimulation or inhibition of specific brain areas can alter dysfunctional neural circuits and restore balance in brain activity, representing a promising alternative for patients who do not respond adequately to conventional treatments or who experience significant side effects [41,42]. They can be divided into two categories: (1) Invasive: Deep Brain Stimulation (DBS) is widely used in the treatment of patients who do not respond to pharmacological treatments for conditions such as Parkinson’s disease, obsessive–compulsive disorder, and headaches [43,44]. (2) Non-invasive: These techniques, along with cognitive stimulation, allow cortical excitability to be modified without resorting to surgical procedures or the use of medications [35]. This group includes Transcranial Magnetic Stimulation (TMS), Electroconvulsive Therapy, or Transcranial Direct Current Stimulation (tDCS). The latter is distinguished by its ease of use, lack of pain, scarcity of significant side effects, and reduced cost [45]. The tDCS is a technique that uses low-intensity electrical currents to modulate neuronal activity in specific brain regions [46,47]. The anode hyperpolarizes the membranes of cortical neurons, which increases their excitability, while the cathode depolarizes them and inhibits functional circuits. In this way, direct current modulates the rate of spontaneous discharge in neural networks, either exciting or inhibiting them [48]. By combining the application of currents with cognitive training aimed at a specific cognitive function, the effects of neural plasticity are enhanced, resulting in broader and more lasting effects [49]. Electrode placement on the head is a critical factor to consider, as the effects of stimulation can vary depending on whether the entire brain or specific regions of the cerebral lobes are stimulated [50,51]. In patients with ADHD, transcranial direct current stimulation (tDCS) has led to improvements in the imbalance between hypoactivity of the left dorsolateral prefrontal cortex (DLPFC) and hyperactivity of the right DLPFC, which are primarily responsible for cognitive and emotional regulation [39,52]. This causes changes in the glutamatergic system and the GABA neurotransmitter, which impact brain neurotrophic factors and affect neural plasticity. Ironside et al. [53] showed that after applying anodal stimulation via tDCS to patients with high anxiety levels over the dorsolateral prefrontal cortex, amygdala response was reduced, and consequently, anxiety and depression decreased to a low level. Another meta-analysis by Razza et al. [54] concluded that patients who received active tDCS obtained greater improvements in depressive symptoms than those who received placebo.

Despite the growing interest in using tDCS as a non-invasive neuromodulation method for ADHD and other cognitive disorders, research in adults remains limited. No established protocols currently integrate tDCS with both digital cognitive rehabilitation and psychotherapy. Systematic reviews focusing on adults with ADHD highlight the scarcity of studies combining tDCS with cognitive stimulation tasks or emotional regulation components. The existing evidence is primarily limited to executive functions such as attention and inhibitory control, and it lacks protocols that address combined interventions across multiple therapeutic modalities [39]. Moreover, while several tDCS trials have explored its effects on cognitive measures in ADHD, most research has focused on tDCS as a standalone intervention or in pediatric populations. Clinical benefits on core symptoms in adults remain inconclusive [55].

This single-case study serves as a feasibility and pilot investigation intended to provide preliminary insights into the feasibility of an integrated protocol combining tDCS, digital cognitive rehabilitation, and psychotherapy for adult ADHD. Rather than establishing definitive efficacy, our goal was to explore the clinical viability and safety of this multimodal protocol in a real-world setting. While we hypothesized improvements in attention, executive functions, and clinical symptoms, these findings are intended to serve as pilot-level evidence to encourage future controlled investigations into the synergies of neuromodulation and behavioral interventions in the underrepresented adult ADHD population.

## 2. Materials and Methods

### 2.1. Design

A single-case experimental design A-B was used in this study [56]. The treatment consisted of two main phases: (1) an initial baseline phase without therapy, and (2) a therapy phase.

### 2.2. Study Subject

A 21-year-old right-handed single woman of Spanish nationality, pursuing a degree, attended a University Psychology Clinic. The patient has an older sister and lives with her parents. She was diagnosed with predominantly inattentive ADHD during childhood. She received psychoeducational intervention during school years but never followed a specific psychological o pharmacological treatment. The patient sought consultation due to academic concerns, lack of motivation, frustration, and various personal problems. Upon arrival at the University Clinic, the diagnosis was confirmed by a certified specialist in clinical psychology based on a thorough clinical examination following DSM-5-TR criteria [1], integrating developmental history and multi-source clinical data to ensure a robust differential diagnosis. Her participation in the study was voluntary and non-randomized, providing informed consent. The patient’s neurological status was screened via clinical history to ensure the absence of comorbidities. Inclusion criteria required no history of neurological disorders (e.g., seizures), no metallic implants, and no medical devices susceptible to electrical interference, no presence of substance abuse and not being pregnant or lactating. No additional neurological tests, such as neuroimaging or EEG, were performed as part of this feasibility study.

### 2.3. Instruments

The following instruments were used to assess cognitive performance and emotional state before and after treatment:

#### 2.3.1. Cognitive Assessment

Nesplora Aquarium virtual reality test: This test uses virtual reality to conduct a continuous dual-task assessment of attention, inhibitory control, and impulsivity [57]. It involves changing the target stimulus (e.g., fishes or their names). In the learning task, the person must respond whenever they see or hear the target stimulus, while in another part, the subject must respond to everything except when the target stimulus appears. It measures omission errors (sustained attention), commission errors (inhibitory control), and reaction time in commissions (impulsivity).

Letter–Number Sequencing subtest (WAIS-IV): A subscale of the Wechsler Adult Intelligence Scale that evaluates auditory working memory (WM). The patient must order the letters heard from the examiner alphabetically and the numbers heard from the examiner in ascending order. Letters must be stated first, then numbers [58].

Spatial Span subtest (WMS-III): A subscale of the Wechsler Memory Scale-III that assesses visuospatial working memory. The examinee is presented with 10 cubes heterogeneously arranged on a board. The patient does not see the numbers behind each cube, which are only visible to the examiner as a guide. The examiner taps the cubes, and the examinee must repeat the movements, first directly and then in reverse. This subtest is used to evaluate visuospatial working memory [59].

Five-Digit Test (FDT): This test employs the same principle as the Stroop test, but with numbers and asterisks instead of words and colors, allowing for greater test variety. It measures processing speed, the ability to cope with interference, and cognitive flexibility. It is measured in seconds of execution, so a higher score means poorer performance [60].

Rings Test: A test designed to evaluate the higher order executive function of planning. It consists of the examinee moving rings one by one, placed by the examiner on a three-post board, to reproduce a model presented on a sheet. It has 15 items. The measurement scale is seconds, so the more seconds taken, the worse the performance in the task [61].

#### 2.3.2. Emotional Assessment

PHQ-9 (Patient Health Questionnaire-9): The tool consists of nine questions used to assess depressive symptoms, covering various areas such as mood, interest in daily activities, sleep, appetite, energy, and concentration. Its main purpose is to determine the presence and intensity of depressive symptoms. Each question is scored based on the frequency of symptoms, and the total score ranges from 0 to 27, which helps assess the severity of depression. In this questionnaire, the results are measured inversely, meaning that a higher score indicates a greater risk of severe depression [62]. The clinical severity is categorized into five levels:

1. Score range 1–4: None or minimal symptoms
2. Score range 5–9: Mild symptoms
3. Score range 10–14: Moderate symptoms
4. Score range 15–19: Moderate–severe symptoms
5. Score range 20–27: Severe symptoms

GAD-7 (Generalized Anxiety Disorder-7): A seven-question questionnaire used to assess anxiety symptoms, exploring areas such as restlessness, excessive worry, and physical symptoms related to anxiety. Each question is scored according to the frequency and severity of symptoms, and a total score is obtained that helps determine anxiety severity (min. 0, max. 21). The higher the patient’s score, the worse the perceived anxiety symptoms. Although it does not provide a definitive diagnosis, the GAD-7 is useful in assessing and monitoring anxiety symptoms [63]. The clinical severity is categorized into four levels:

1. Score range 0–4: No anxiety symptoms
2. Score range 5–9: Mild symptoms
3. Score range 10–14: Moderate symptoms
4. Score range 15–21: Severe symptoms

WHOQOL-BREF (World Health Organization Quality of Life-Abbreviated): A tool used to assess health-related quality of life (QoL). It consists of 26 questions exploring general QoL and health (2 items), and four domains: physical health (7 items), psychological health (6 items), social relationships (3 items), and environmental aspects (8 items). Each domain has specific questions, and responses are rated on a 1–5 scale. The questionnaire provides a global measure of quality of life (min. 26, max. 130), with higher scores showing a higher QoL, and is widely used in research [64].

tDCS-related sensations questionnaire: This self-assessment questionnaire is used to determine if the patient experiences any strange or unpleasant sensations after receiving transcranial direct current stimulation (tDCS). It allows for measuring the intensity of these sensations on a Likert scale, where scores are assigned from “none” to “strong” (0–3) [65]. This questionnaire helped to determine if at any point the intervention needed to be stopped due to side effects produced by tDCS in the patient. It was administered after each session.

### 2.4. Procedure

**Figure 1:**
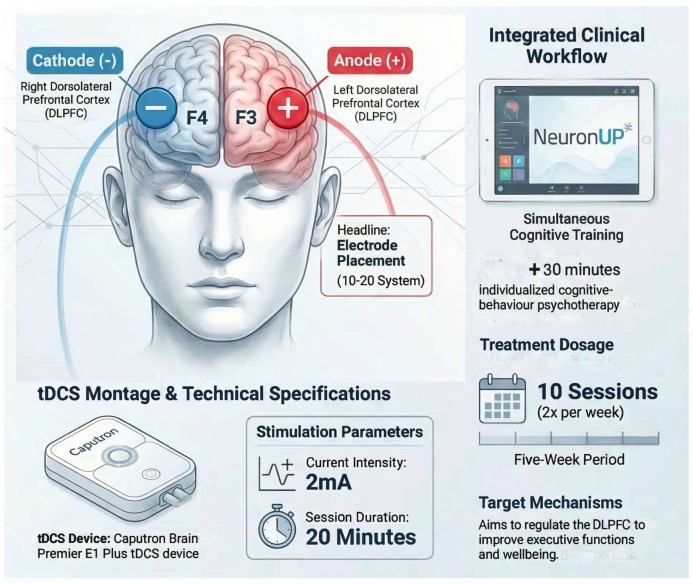
Multimodal Neuromodulation Protocol for Adult ADHD.

The patient was attended by a certificated clinical neuropsychologist at a University Psychology Clinic. The procedure was divided into the following stages:

1. **Initial interview:** The details and conditions of the study were explained. Once the Information Sheet was read and understood, an Informed Consent was then voluntarily signed.
2. **Pre-Intervention assessment:** A semi-structured questionnaire was used to collect demographic data and relevant clinical parameters. Then, cognitive and emotional evaluations were performed to obtain the baseline performance.
3. **Intervention Phase:** Three main instruments were used during the five weeks of treatment: digital cognitive stimulation, tDCS neuromodulation, and psychotherapy. The treatment consisted of a combination of cognitive stimulation using exercises on the “NeuronUp” neurorehabilitation platform [66] along with transcranial direct current stimulation (tDCS), using a Caputron Brain Premier E1 Plus tDCS device (Registration No: DEBB104012088) with anodal current of 2 mA on the left dorsolateral prefrontal cortex (F3), while the cathode was placed on the right dorsolateral prefrontal cortex (F4). These specific points were selected due to their ability to activate the dorsolateral prefrontal cortex (DLPFC) and regulate the imbalance between both hemispheres [39]. Each session, offered twice a week, lasted a total of 60 min. First 30 min were used for tDCS setup, 20 min of active neuromodulation treatment while performing cognitive exercises in NeuronUP and finally discussing perceived tDCS sensations. Each cognitive stimulation session had a total of four interactive exercises, each lasting five minutes. The difficulty levels of each activity were regulated according to the patient’s performance, utilizing the platform’s built-in ‘staircase’ algorithm. This system automatically increases task complexity (e.g., number of stimuli) after three consecutive successes and decreases it after two failures, maintaining the task within the patient’s optimal challenge zone [66]. A program with interactive, dynamic, and engaging online activities was created. Attention, working memory, processing speed, inhibitory control, and planning functions were stimulated. In addition, the patient received 30 additional minutes of individualized cognitive–behavior psychotherapy that focused on identifying and modifying dysfunctional thinking patterns and behaviors, helping the patient to develop healthier cognitive patterns and enhance coping strategies through structured interventions, including thought records, behavioral experiments, and skills training. The specific electrode placement and the integrated treatment flow, including the simultaneous application of tDCS and digital cognitive exercises plus CBT, are illustrated in Figure 1.
4. **Post-Intervention assessment:** Cognitive and emotional evaluations were carried out again to determine if cognitive performance and emotional state had improved.

### 2.5. Statistical Analysis

The different tools used to analyze the obtained data are described below.

Psycho R package (v.0.6.1): This package is designed to facilitate statistical analysis and the preparation of documents ready for publication in the fields of psychology, neuropsychology, and neuroscience [67]. In this study, the ‘Psycho’ package was used to perform the analysis of the Crawford and Garthwaite index and the Mellenbergh and van de Brink significance index.

Crawford and Garthwaite index [68]: Used in single-case studies in neuropsychology, this index allows to determine if the observed difference in the patient is significantly different from that of the control group sample. Besides providing a hypothesis test, it also offers a point and interval estimate of the abnormality of the difference between the case scores. This is particularly useful when control samples with which a patient is compared generally have a small N. The method consists of repeatedly applying an equation that involves the scores obtained by the patient in each of the assessment tests, as well as the mean and standard deviation of a control sample. This allows determining the subject’s distribution before and after the intervention, represented by the value known as Zcc. From this value, the ‘Zcc’ index provides the lower and upper limits of a 95% credibility interval for the true effect size.

The index developed by Mellenbergh and van de Brink [69] provides a measure of individual differences before and after treatment, allowing for the evaluation of both the magnitude and significance of the observed change. This index allows addressing the limitations of repeated testing by evaluating individual progress while accounting for measurement error. It is important to note that since this was a single-case design, the ‘control group’ data used in the Crawford and Garthwaite, and Mellenbergh and van den Brink indices referred exclusively to normative samples provided by the tests’ manuals and previous validated studies. To further analyze intrasubject changes in clinical significance, determined by a change in the participant’s clinical classification and a shift of at least 1 Standard Deviation (SD) relative to the pre-test performance level, we followed the Rodríguez-Prieto et al. [70] procedure based on Korkman et al. [71] recommendations. The qualitative categories and clinical ratings can be seen in the Appendix A, Table A1. This conservative Reliable Change criterion ensured that only improvements surpassing typical measurement fluctuations were categorized as clinically meaningful.

SPSS (v.28): The Statistical Package for the Social Sciences, also known as SPSS, is a statistical program widely used in research and data analysis. In single-case studies, the Wilcoxon index is a non-parametric statistical test used to compare the scores obtained by a single person before and after intervention to determine if there are significant differences between them or if they are due to chance [72]. This aggregate analysis of heterogeneous measures is justified in N-of-1 feasibility frameworks as a means to evaluate the overall consistency and direction of clinical gains across different domains, treating the set of standardized pre-post scores as related samples to determine if the global improvement trend is statistically reliable. It is used when data do not follow a normal distribution, making it particularly useful in research with small samples or with outliers. The result of the Wilcoxon test consists of a test statistic called “z” and an associated *p*-value. If the *p*-value is less than a significance level of 0.05, it is concluded that there is sufficient evidence to state that there is a significant difference between the two related samples. We acknowledge that this aggregate analysis combines heterogeneous measures; therefore, it is interpreted solely as a measure of global clinical trend within this feasibility framework.

## 3. Results

The results of the pretest and posttest for each assessment tool were analyzed to determine the difference between the individual and the control sample, typically obtained from the normative data of the tests (Crawford and Garthwaite index), as well as the magnitude and observed change between these two measures and their level of significance (Mellenbergh and van de Brink index). Subsequently, the differences in the total set of results from all cognitive and emotional measures were analyzed to compare pretest and posttest results, allowing us to determine if the differences were significant or due to chance (Wilcoxon index). Finally, the analysis of changes in clinical significance was used to determine if the treatment was effective and to what extent.

### 3.1. Cognitive Performance

Table 1 presents a summary of main results obtained in cognitive measures in the pre- and post-intervention assessments.

**Figure 2:**
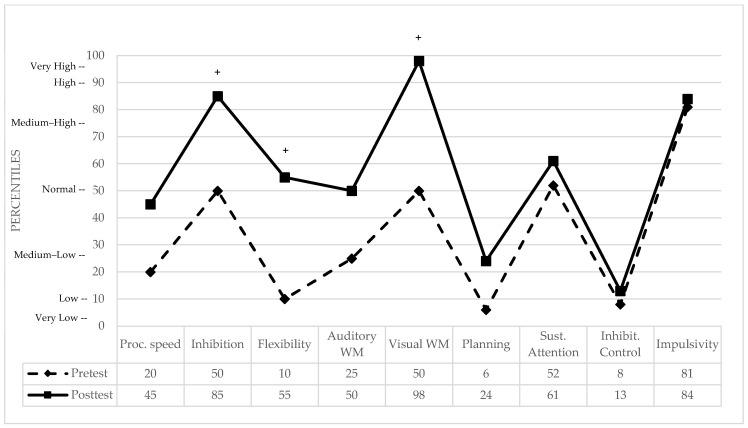
Pre- and post-treatment cognitive performance in percentiles. WM = Working Memory. + indicates a clinically significant difference between pre- and post-test performance.

Five-Digit Test: Three indices were analyzed, Reading (processing speed), Inhibition (interference control), and Flexibility. Performance was compared against a normative control group of Spanish adults [60]. While the statistical indices Crawford and Garthwaite, and Mellenbergh and van den Brink, did not reach significance thresholds (see Table 1), clinically relevant improvements were observed. Most notably, in Cognitive Flexibility, the patient moved from the 10th percentile (slight clinical deficit) to the 55th percentile (normal range). Similar positive clinical shifts were found in processing speed (Reading) and interference control (Inhibition), with the patient reaching the 45th and 85th percentiles, respectively (see Figure 2 for pre- and post-treatment performance in percentiles and clinically significant differences).

Letter–Number Sequencing: No significant differences were observed between the patient and the normative control sample [58] in either the pretest or posttest (see Table 1). Furthermore, the Mellenbergh and van den Brink index confirmed the absence of a significant intervention effect for this auditory working memory measure (*p* = 0.520). While the patient’s performance improved from the 25th to the 50th percentile, both scores remain within the clinically normal average range (see Figure 2).

Spatial Span: At baseline, the patient’s performance was identical to the normative control mean [59] (see Table 1). Following the intervention, she showed a statistically significant superior performance compared to the normative control group (*p* = 0.015). While the Mellenbergh and van den Brink index did not reach the significance threshold for the intrasubject change, a highly relevant clinical improvement in visuospatial working memory was observed. Specifically, the patient shifted from the 50th percentile (average range) to the 98th percentile, placing her in the ‘very high’ range or ‘high talent’ category (see Figure 2).

Rings Test: Evaluation of this higher-order executive function revealed a meaningful transition from impaired to normalized planning performance. At baseline, the patient’s score reflected low performance, bordering on statistical significance compared to the normative control group (see Table 1). Following the intervention, her execution time decreased, showing no significant difference from the normative mean [61]. While the Mellenbergh and van den Brink index did not reach the statistical significance threshold for the intrasubject change, a modest clinical improvement (<1 SD) was documented. Specifically, the patient moved from the 6th percentile (low; small deficit) to the 24th percentile (medium–low; clinically normal). Although this shift remained below the 1 SD threshold defined for ‘clinically significant change’ in this study, the findings reflect a normalization of complex planning abilities, as the patient’s performance at post-test fell within the normative range of the healthy population (see Figure 2).

Nesplora Aquarium Virtual Reality Test: Since the digitalized test only provided standardized results automatically, and neither the mean values nor the standard deviations of the control sample were reported, the Crawford and Garthwaite index and the Mellenbergh and van den Brink index could not be applied. Therefore, only the intrasubject changes in clinical significance were analyzed (see Figure 2).

- Sustained attention. In this measure of omission errors, the patient obtained a normal performance, positioning herself in the 52nd percentile in the pretest results, with little variation in the posttest score, placing her in the 61st percentile, where her performance remains in a normal range.
- Inhibitory control. At baseline, the patient’s performance in commission errors was in the 8th percentile, reflecting a clinically small deficit in prepotent response inhibition. Following the intervention, performance showed a marginal shift to the 13th percentile (medium–low range). Despite this slight improvement, her post-treatment performance remains classified as a clinically small deficit in the ability to inhibit motor responses under the high cognitive load of a virtual reality dual-task.
- Impulsivity. In this measure the patient obtained a medium–high performance in the pretest, placing her in the 81st percentile (clinically normal). In the posttest, she maintained a medium–high performance, positioning herself in the 84th percentile.

Finally, a comparison was made between all the evaluations performed in the pretest and posttest to determine whether the intervention was effective as a whole, rather than focusing on specific cognitive functions. Analyzing the set of scores across the different assessment measures using the Wilcoxon index, a clinical significance was observed (*Z* = 2.67, *p* = 0.008). This indicates that the differences between pre-intervention and post-intervention were not due to chance and can be considered a genuine improvement in cognitive performance following the combined treatment.

### 3.2. Emotional State

Table 2 presents a summary of emotional and wellbeing measures in the pre- and post-intervention assessments.

PHQ-9: Regarding depressive symptoms, the patient’s baseline profile was significantly different from the normative population [73], categorized as moderately severe depression. Following the intervention, these scores normalized, showing no significant difference relative to the control group (see Table 2). Clinically, the patient demonstrated a meaningful improvement, moving from a moderately severe level—where combined medication and therapy are typically recommended—to a moderate-mild level, where psychological treatment alone is recommended.

**Figure 3:**
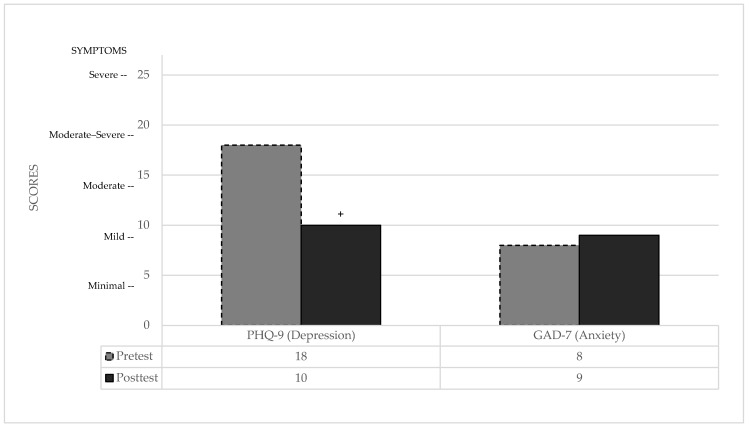
Pre- and post-treatment emotional state scores. + indicates a clinically significant difference between pre- and post-test performance.

GAD-7: In contrast, anxiety levels remained stable and showed no significant differences compared to the normative control group [73] in either assessment (see Table 2). The patient remained within the range of mild anxiety symptoms throughout the study, indicating no clinically significant change in this domain (see Figure 3).

**Figure 4:**
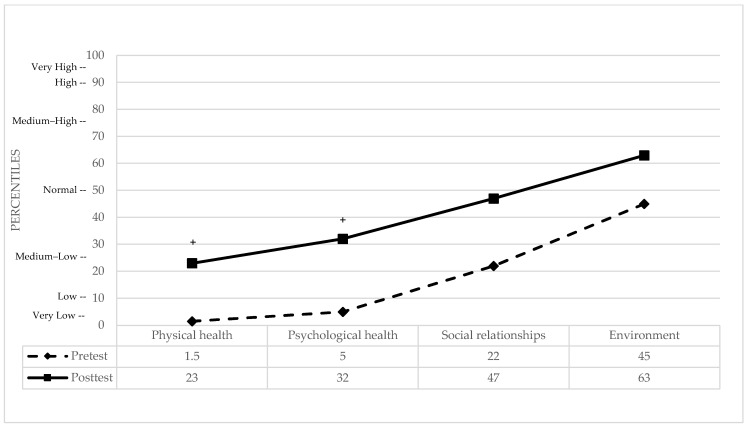
Pre- and post-treatment domains of QoL in percentiles. + indicates a clinically significant difference between pre- and post-test performance.

WHOQOL-BREF: The four domains of Quality of Life (QoL) were analyzed, with comprehensive statistical indices summarized in Table 2. Significant clinical improvements were observed in Physical and Psychological health against a normative control group [74]. In the Physical domain, the patient moved from a very low percentile (representing a significant baseline deficit) to a medium–low percentile, reflecting a notable improvement in perceived physical well-being. Similarly, in Psychological health, the patient shifted from the 5th percentile (moderate deficit) to the 32nd percentile, successfully reaching the clinically normal range. In contrast, the Social relationships and Environment domains remained stable throughout the study. In both cases, the patient’s baseline perception was already within the clinically normal range (22nd and 45th percentiles, respectively) and showed further shifts toward higher average levels (47th and 63rd percentiles) following the intervention. Overall, the multimodal treatment appeared most effective in normalizing the QoL domains that were initially impaired (see Figure 4).

Finally, the effectiveness of the intervention was assessed by comparing all emotional state measures from the pretest and posttest. The results indicated that the differences between pre-intervention and post-intervention scores were unlikely to be due to chance, showing a statistically significant difference (*p* = 0.046) when analyzing the set of scores, according to the Wilcoxon index.

### 3.3. tDCS-Related Sensations Questionnaire

Throughout the intervention, the questionnaire developed by Antal et al. [65] was administered after each tDCS session to assess potential adverse effects. The patient did not report symptoms such as burning sensation, metallic taste, or fatigue in any of the ten sessions. The most frequently reported symptom was localized itching (40% of sessions), which typically occurred at the beginning of stimulation. On only one occasion did the itching persist throughout the entire session; in all other instances, it resolved during the first half of the session.

## 4. Discussion

The primary objective of this feasibility study was to explore the preliminary outcomes of a multimodal intervention combining anodal tDCS over the dorsolateral prefrontal cortex, digital cognitive stimulation, and psychotherapy in an adult diagnosed with predominantly inattentive ADHD. The aggregate Wilcoxon analysis indicated that the overall improvement across cognitive (*p* = 0.008) and emotional (*p* = 0.046) measures was statistically significant. While these results suggest a positive trajectory following the intervention, they should be interpreted with caution given the heterogeneity of the tests. This aggregate finding serves to highlight the consistency of the clinical gains observed across the multimodal protocol, suggesting that it could successfully modulate a patient’s neurocognitive and emotional profile. Nevertheless, due to the A-B single-case design, it should be interpreted as pilot-level evidence within a feasibility framework rather than definitive proof of treatment effectiveness.

### 4.1. Impact on Cognitive Performance and Executive Functions

Regarding cognitive outcomes, the patient demonstrated clinically significant improvements in higher-order executive functions, specifically in planning, cognitive flexibility, and visual working memory. The improvement in planning (Rings Test) was particularly noteworthy, as the patient moved from a low-performance range to the normal range, as detailed in Table 1, following the 10-session protocol. This finding aligns with evidence suggesting that stimulation of the left dorsolateral prefrontal cortex (DLPFC) enhances the synaptic plasticity necessary for complex executive tasks [39]. At the cellular and molecular levels, the interaction between tDCS and neuropsychological treatment is hypothesized to be synergistic. Anodal tDCS over the left DLPFC may facilitate a state of cortical excitability that lowers the threshold for neuronal firing, potentially inducing Long-Term Potentiation [75]. This ‘priming’ effect may increase the expression of brain-derived neurotrophic factor (BDNF), which in turn enhances synaptic plasticity during the performance of cognitive tasks [76]. Consequently, neuropsychological training provides the task-specific ‘signal,’ while tDCS optimizes the ‘neural soil,’ improving the efficiency of the executive networks. This interaction is particularly relevant in the context of adult ADHD, a condition influenced by multiple genetic variants that affect catecholaminergic signaling [12,13]. While our clinical findings are consistent with this model, they remain exploratory in nature. This case study provides preliminary insights into how prefrontal modulation might optimize the ‘neural soil’ for cognitive tasks, but these mechanistic pathways require verification through neurophysiological monitoring in future trials. Unlike pediatric ADHD, where treatment often targets active neurodevelopmental maturation and cortical thickening, intervention in adults aims to modulate stabilized but hypoactive prefrontal-striatal circuits. Given the polygenic nature of the disorder, the efficacy of combined protocols may vary based on individual genetic profiles, suggesting that in adults, the treatment acts as a compensatory framework for executive dysfunction rather than a purely corrective developmental intervention.

No significant changes were observed in sustained attention and impulsivity (Nesplora Aquarium), or auditory working memory (Letter–Number Sequencing). This lack of improvement may be partially explained by a ceiling effect, as the patient’s baseline performance in sustained attention and impulsivity was already within the normal range. The absence of observed improvements in auditory working memory could also be attributed to the patient’s individual characteristics, such as differences in brain anatomy or genetic factors [77]. Furthermore, the intensity of the electric current used in transcranial stimulation can spread to broader brain areas, making precise targeting of the dorsolateral prefrontal cortex challenging. The response to stimulation may also depend on factors such as the participant’s mental and physical health, age, or prior exposure to stimulation, all of which can influence the efficacy of tDCS [78]. Consequently, the specific tDCS configuration—including parameters such as intensity (mA), duration (in this case, ten sessions of 20 min each), and electrode placement (targeting the dorsolateral prefrontal cortex)—can also influence the outcomes [79]. These factors may be related to the intriguing dissociation observed in inhibitory control. While the Five-Digit Test (FDT) demonstrated a clinical improvement in interference control, the commission error rate in the Nesplora Aquarium test remained in the deficit range. This discrepancy may be attributed to the differing cognitive demands of the tasks: the FDT measures the speed of processing interference in a structured environment, whereas the Aquarium test requires response inhibition under a high cognitive load (dual-task) in a virtual reality setting. This distinction aligns with the differentiation proposed by Friedman & Miyake [80] between interference control (FDT) and prepotent response inhibition (Aquarium). The absence of gains in the latter could be related to individual neuroanatomical differences or the need for more targeted stimulation to modulate motor-response inhibition [81].

### 4.2. Emotional Regulation and the Confounding Effect of Psychotherapy

The results indicated a robust reduction in depressive symptoms (PHQ-9) and a significant enhancement in psychological quality of life (WHOQOL-BREF). The intervention led to a significant clinical reduction in depressive symptoms and a normalization of the patient’s emotional profile (see Table 2). The reduction in depressive symptoms is theoretically grounded in the model of prefrontal-limbic regulation [53]. It is hypothesized that anodal stimulation over the DLPFC promotes cortical regulation of limbic structures, such as the amygdala and hippocampus, which are often dysregulated in ADHD. However, in the absence of objective neuroimaging data, these observations should be treated as hypothesis-generating interpretations. The observed improvements suggest a potential clinical shift in emotional regulation, but we cannot conclude a direct mechanistic link between the stimulation and limbic modulation based solely on this case.

However, a critical confounding factor must be addressed: the concurrent administration of cognitive–behavioral psychotherapy. The observed emotional benefits likely represent a synergistic effect where neuromodulation facilitates the biological readiness for change [54,82,83], while psychotherapy provides the behavioral strategies to manage anhedonia and functional impairment. As previously mentioned, tDCS promotes an increase in synaptic plasticity in the human brain, which represents a potential mechanism of action for improving depression. The tDCS applied to the dorsolateral prefrontal cortex influences synaptic plasticity and strengthens neuronal connections in brain areas involved in adaptability and cognitive change [84]. In contrast, anxiety symptoms (GAD-7) remained stable. This lack of response supports the view that anxiety is a multiregional construct involving circuits beyond the reach of a purely DLPFC-focused montage [81,85]. Furthermore, external stressors—specifically the patient’s academic exam period during the post-test—may have maintained anxiety levels, potentially masking any therapeutic gains from the neuromodulation. While external stressors are an unavoidable component of clinical reality, particularly in adult ADHD, they act as constant triggers for emotional responses that can interfere with therapeutic gains [53]. Future research should move beyond static measures by incorporating Ecological Momentary Assessment (EMA) [86]. This methodology allows for the real-time monitoring of the dynamic interaction between environmental demands and anxiety fluctuations, providing a more granular understanding of how tDCS-induced prefrontal modulation translates into daily life resilience. Furthermore, to specifically address the multiregional nature of anxiety, integrating neuromodulation with Mindfulness-Based Stress Reduction (MBSR) or Emotion Regulation Therapy (ERT) is proposed as a synergistic strategy. Meta-analytic evidence suggests that these behavioral interventions can strengthen the ‘top-down’ regulatory window of opportunity created by tDCS, helping patients consolidate long-term coping mechanisms against unavoidable external stressors [87].

Regarding the social relationships and environment domains of the WHOQOL-BREF, no significant clinical improvements were observed. This lack of change likely stems from the fact that these domains are heavily contingent upon external socio-environmental variables and interpersonal dynamics that are not directly modulated by the internal regulatory mechanisms of the DLPFC. While anodal tDCS effectively enhances the cortical regulation of emotional and cognitive processes, its impact on the patient’s objective living conditions or social network remains indirect. Furthermore, although the psychotherapy sessions specifically addressed ‘problems at home’ and ‘relationships with friends’, the stability of these scores reinforces the notion that these aspects of quality of life are inherently more stable or depend on objective environmental shifts that did not occur within the five-week treatment period. Additionally, a ceiling effect must be considered, as the patient’s baseline scores in these areas were already within the clinically normal range, leaving limited margin for further significant gain.

### 4.3. Safety, Tolerance, and Methodological Rigor

In this study, significant attention was given to the information provided by the patient through the tDCS-related sensations questionnaire [65] to assess tolerance to the technique and monitor potential side effects. It can be concluded that the intervention was safe and well-tolerated, with localized itching being the only mild and transient side effect reported, as commonly observed [88]. This supports tDCS as a favorable alternative to the adverse effects often associated with pharmacological stimulants or antidepressants in the adult population [3]. The integrated protocol described here could serve as a framework for other neurocognitive disorders. For instance, in neurodegenerative conditions such as Alzheimer’s disease, where memory loss is paramount, the combined use of anodal tDCS over the DLPFC with cognitive training has been shown to modulate executive networks that support memory retrieval [89]. Similarly, prefrontal stimulation has already shown promise in improving cognitive and emotional function in various conditions, including major depression [90].

Despite the inherent limitations of a single-case experimental design (N = 1), the study’s internal validity is strengthened by the use of robust statistical indices. The application of the Crawford and Garthwaite [68] Bayesian approach and the Mellenbergh and van de Brink index [69] enabled a rigorous comparison of individual changes against normative populations. However, in the Nesplora Aquarium, a digitalized virtual reality test that directly reports the patient’s results in an automated format with standardized scores, these indices could not be applied, as normative reference scores (mean and standard deviation) for the control sample were not available for comparison. Nonetheless, all measures could be analyzed statistically using the Wilcoxon test [72], complemented by significant clinical shifts in percentiles. Regarding the statistical interpretation, it is important to note that while the aggregate Wilcoxon analysis showed significant global progress, individual measures did not reach the significance threshold of the Mellenbergh and van den Brink index. This index is recognized for its high conservative nature, as it strictly accounts for measurement error and the variability inherent in repeated testing. To balance this rigor with clinical reality, we complemented the analysis with a Reliable Change criterion of 1 SD. Consequently, several observed improvements resulted in final scores that, while reflecting a normalization of functions, still remain within average or medium–low ranges. These findings should therefore be discussed conservatively as signs of symptomatic normalization and improved functional capacity rather than total symptom remission. This framework provides compelling emerging evidence for the feasibility of integrated neuromodulation protocols in treating the complex and often underrepresented adult ADHD population. However, we must consider that the efficacy of tDCS is highly sensitive to individual anatomical differences and the optimization of stimulation parameters, as emphasized by Fathipour-Azar et al. [79], so future protocols may need to further tailor intensity and duration to target specific, resistant cognitive and emotional sub-domains. Another limitation of this study is the lack of long-term follow-up. While the standardized measures used possess high sensitivity to detect post-intervention shifts, they do not allow us to determine the longitudinal stability of the observed clinical gains. Future research should implement longitudinal designs to evaluate whether the combined effects of tDCS and neuropsychological treatment persist over several months. The absence of neurophysiological and neuroimaging data is also a limitation of this study. These objective measures are instrumental in confirming the neural mechanisms underlying clinical improvements. Future research following this pilot feasibility study should integrate EEG or fMRI to transition from hypothesis-generating observations to the demonstration of specific mechanistic effects.

Finally, while the global improvements in patients are encouraging, the simultaneous administration of modalities makes it impossible to disentangle the specific weight of tDCS from the effects of psychotherapy or cognitive training. The observed clinical shifts are hypothesized to stem from a synergistic ‘priming’ effect, but we cannot rule out the influence of practice effects or spontaneous symptomatic fluctuations inherent to the A–B design. Consequently, a primary limitation of this study is the lack of a sham-controlled condition or a reversal phase, which limits our ability to make causal claims. Future investigations must utilize multiple baseline designs or randomized sham-controlled trials to rigorously isolate the neuromodulatory effect from the behavioral components.

## 5. Conclusions

The hypothesis of this study has been partially fulfilled, as the combined treatment of cognitive stimulation and psychotherapy with transcranial direct current stimulation (tDCS) in the dorsolateral prefrontal cortex has generated positive emotional results (depression and physical and psychological quality of life) and in several cognitive abilities (inhibition, flexibility, visual working memory, and planning) in a patient with adult ADHD. No significant improvements were observed in anxiety, QoL related to social relationships or the environment, processing speed, auditory working memory, or sustained attention.

This single-case study, while not generalizable, can serve as a pilot study for future controlled trials with a larger number of participants, more extended intervention periods, and longitudinal evaluations. tDCS is presented as a rapid, simple to administer, cost-effective, and safe treatment due to the low incidence of side effects, which makes it a favorable alternative to the typical side effects of antidepressants. Nevertheless, tDCS should be considered a complementary and not an independent treatment, to be used in combination with other therapies to maximize its efficacy.

## Figures and Tables

**Table 1:** Summary of cognitive results.

Variable	Pre-Score	Post-Score	Crawford *p* (Pre)	Crawford *p* (Post)	Mellenbergh *p*
FDT. Reading	21 s	18 s	0.250	0.550	0.250
FDT. Inhibition	12 s	7 s	0.457	0.146	0.488
FDT. Flexibility	29 s	20 s	0.125	0.443	0.361
Letter–Number Seq. (WM)	5	6	0.361	0.296	0.520
Spatial Span (WM)	17	23	0.480	0.015	0.123
Rings (Planning)	278 s	239 s	0.087	0.890	0.521

Note: FDT = Five-Digit Test, WM = Working Memory, s = seconds.

**Table 2:** Summary of emotional and wellbeing results.

Variable	Pre-Score	Post-Score	Crawford *p* (Pre)	Crawford *p* (Post)	Mellenbergh *p*
PHQ-9 (depression)	18	10	0.025	0.254	0.355
GAD-7 (anxiety)	8	9	0.219	0.157	0.868
WHOQOL. Physical	44	63	0.014	0.227	0.303
WHOQOL. Psychological	38	63	0.046	0.316	0.395
WHOQOL. Social	56	69	0.367	0.452	0.611
WHOQOL. Environment	69	75	0.445	0.366	0.735

## Data Availability

The data supporting the findings of this study are available upon request from the corresponding author due to privacy reasons.

## Abbreviations

The following abbreviations are used in this manuscript:

ADHD	Attention Deficit Hyperactivity Disorder
CBT	Cognitive–Behavioral Therapy
DLPFC	Dorsolateral Prefrontal Cortex
EFs	Executive Functions
FDT	Five Digit Test
QoL	Quality of Life
tDCS	Transcraneal Direct Current Stimulation
TMS	Transcraneal Magnetic Stimulation
WM	Working Memory

## Appendix A

Changes in Clinical Significance: Starting from the 50th percentile (average), a series of three consecutive improvements, each with a magnitude of 1 standard deviation (SD), would result in the patient being reclassified as “medium–high,” “high,” and “very high,” respectively. Similarly, a series of three consecutive reductions, each with a magnitude of 1 SD, would reclassify the patient as “medium–low,” “low,” and “very low,” respectively. Therefore, when comparing the case to her reference group, we defined a clinical improvement as a change of at least 1 SD (either above or below the pretest performance level).

**Table A1:** Qualitative description and clinical significance of percentile scores (PC) and standard deviations (SD). Based on Korkman et al. [71] and Rodríguez-Prieto et al. [70].

PC (Range)	SD	Description	Clinical Significance
99.9	3.0		
99.6	2.7	Very High	Extremely talented
98–99	2.3		
95–97	2.0		Highly talented
90–94	1.7	High	
85–89	1.3		Talented
76–84	1.0	Medium–High	
63–75	0.7		
51–62	0.3		
50	0	Average	Normal
38–49	−0.3		
26–37	−0.7		
17–25	−1.0	Medium–Low	
11–16	−1.3		Small deficit
6–10	−1.7	Low	
3–5	−2.0		Moderate deficit
1–2	−2.3		
0.4	−2.7	Very low	Severe deficit
0.1	−3.0		

Note. PC = Percentile, SD = Standard Deviation. The color coding in this table is used to visually delimit the qualitative categories of performance interpretation (Description) and to highlight the thresholds for Clinical Significance.
